# Smoking

**Published:** 1868-09

**Authors:** 


### SMOKING

Is a useless, expensive, selfish and filthy practice ; it leads to drunkenness in many cases, and it is rare to find a drunkard who does not smoke ; the man who smokes every day is never safe from the gutter ; and he who deliberately runs this risk, has not the courage to avoid any other sink of moral degradation, were it not for the fear of being found out.

As to the chicken-hearted plea, "I can't quit it," even when convinced that it is wrong and unhealthful, hear the testimony of *James Parton* who was a slave to the practice for thirty years, and who heroically broke from his chains, on the instant of his resolution to do so, "I have less headache, I enjoy exercise more, and step out much more vigorously. My room is cleaner, I think I am better tempered, as well as more cheerful and satisfied. I endure the inevitable ills of life with more fortitude, and look forward more hopefully to the coming years. It did not pay to smoke ; but it decidedly pays to stop smoking."

